# Toward an Understanding of Extracellular tRNA Biology

**DOI:** 10.3389/fmolb.2021.662620

**Published:** 2021-04-15

**Authors:** Adrian Gabriel Torres, Eulàlia Martí

**Affiliations:** ^1^Institute for Research in Biomedicine (IRB Barcelona), The Barcelona Institute of Science and Technology, Barcelona, Spain; ^2^Departament de Biomedicina, Facultat de Medicina i Ciències de la Salut, Institut de Neurociències, Universitat de Barcelona, Barcelona, Spain; ^3^Centro de Investigación Biomédica en Red sobre Epidemiología y Salud Pública, Madrid, Spain

**Keywords:** tRNA, tRNA fragments, extracellular vesicles, regulation of gene expression, cell-to-cell communication, tRNA modifications

## Abstract

Extracellular RNAs (exRNAs) including abundant full length tRNAs and tRNA fragments (tRFs) have recently garnered attention as a promising source of biomarkers and a novel mediator in cell-to-cell communication in eukaryotes. Depending on the physiological state of cells, tRNAs/tRFs are released to the extracellular space either contained in extracellular vesicles (EVs) or free, through a mechanism that is largely unknown. In this perspective article, we propose that extracellular tRNAs (ex-tRNAs) and/or extracellular tRFs (ex-tRFs) are relevant paracrine signaling molecules whose activity depends on the mechanisms of release by source cells and capture by recipient cells. We speculate on how ex-tRNA/ex-tRFs orchestrate the effects in target cells, depending on the type of sequence and the mechanisms of uptake. We further propose that tRNA modifications may be playing important roles in ex-tRNA biology.

## Introduction

Virtually all cells are able to release RNA to the extracellular space [extracellular RNAs (exRNA)] either free, encapsulated in extracellular vesicles (EVs) (Fabbiano et al., 2020; Zietzer et al., 2020), or forming part of complexes with proteins such as Argonaute 2 (Ago2) (Arroyo et al., 2011; Turchinovich et al., 2011) and high-density lipoprotein (HDL) particles (Vickers et al., 2011). ExRNAs can reach other cells, be internalized, and regulate gene expression, even in distant tissues and are thus used for cell-to-cell communications (Valadi et al., 2007; Thomou et al., 2017). Additionally, exRNA content within EVs has been proposed to reflect the active status of the cells of origin (Sadik et al., 2018). Given that transcriptional perturbations, including altered levels of expression, occur generally early in human disease, even before obvious clinical symptoms are detected; exRNA profiling in biofluids is considered a promising strategy for disease diagnosis and prognosis through minimally invasive liquid biopsy.

Studies on the biological significance of exRNA have largely focused on EVs content, which is protected from extracellular RNases. Nevertheless, this concept should be re-examined since the vast majority of exRNA is extra-vesicular and can also be protected from degradation through its binding to proteins or the formation of secondary structures, resistant to nuclease activity (see below). In this sense, the relevance of the most abundant extra-vesicular exRNA is starting to be explored (Vickers et al., 2011; Tosar et al., 2020).

Extracellular RNA is strongly enriched in tRNA and tRNA fragments (tRFs) compared with other species such as microRNAs (miRNAs) that have traditionally received more attention. This has been extensively documented in multiple biofluids such as urine, blood serum, saliva, or cerebrospinal fluid. Thus, these species offer an important source of biomarkers that can sense the biogenesis of tRFs linked to stress and disease (Dhahbi et al., 2013; Zhang et al., 2014; Godoy et al., 2018). The strong abundance of vesicular and non-vesicular extracellular tRNAs and tRFs (ex-tRNA/ex-tRF) has been also confirmed in cell culture media, in diverse *in vitro* studies (Guzman et al., 2015; Tosar et al., 2015; Wei et al., 2017). Several aspects of ex-tRNA/ex-tRF biology are still to date controversial, primarily due to technical issues and limitations that can compromise the interpretation of findings. We refer the reader to a recent and thorough review by Tosar and Cayota (2020) that compiles the current knowledge on the ex-tRNA/ex-tRF field in light of this matter.

**FIGURE 1:**
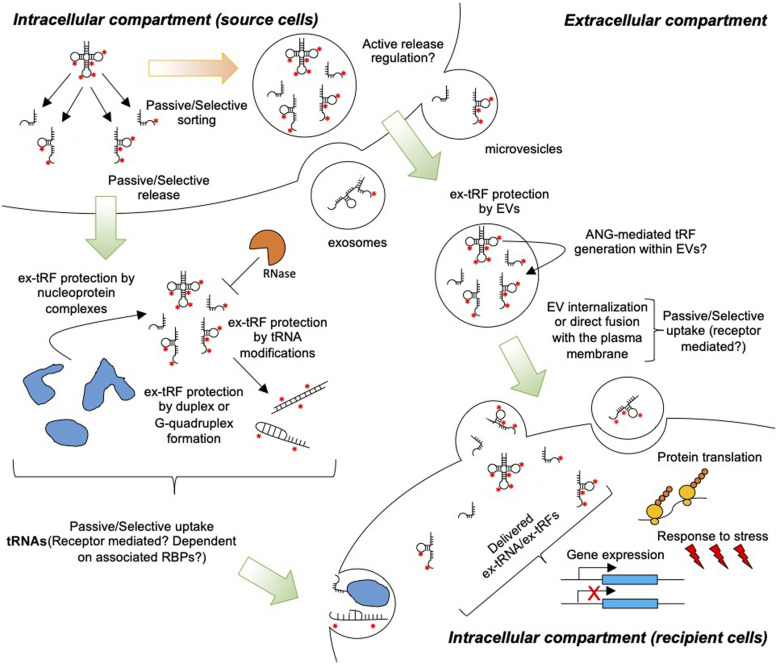
Extracellular tRNA and tRFs in cell-to-cell communication. tRNAs and tRFs are passively or selectively loaded into EVs and released to the extracellular space. Additionally, cells release ex-tRNAs/ex-tRFs that are not associated with EVs, but these species may be protected from degradation by RNases through the formation of secondary structures, G-quadruplexes, and/or ribonucleoprotein complexes. Red asterisks indicate modified nucleotides in tRNAs. Uptake by target cells may occur through the direct fusion of EVs with the plasma membrane, non-specific endocytosis, or receptor-mediated endocytosis. Naked ex-tRNA/ex-tRFs can also be passively or selectively captured by cells. Inside cells ex-tRNAs/ex-tRFs may influence gene expression and/or protein translation, modulating stress and immune responses.

With the realization of non-canonical tRNA functions mediated by either full length tRNAs or tRFs (Avcilar-Kucukgoze and Kashina, 2020), there is a pressing need to further improve our understanding on the generation, release, stability, and uptake of ex-tRNAs and ex-tRFs (Figure 1). Here, we propose that the activity of ex-tRNA and ex-tRFs depends upon the mechanisms of release and capture of these molecules. We reason that tRNA/tRF release is a complex phenomenon, involving cell dependent passive and/or selective RNA sorting in different extracellular compartments, and likewise, that the possible mechanisms of capture and activity of these species in recipient cells may be cell type-specific and depend on the nature of the ex-tRNA/ex-tRF sequences. We further emphasize on the potential roles of post-transcriptional tRNA modifications in ex-tRNA/ex-tRF biology.

## tRNAs and tRFs Are Abundant Components of the Extracellular Compartment

The release of tRNAs and/or tRFs to extracellular compartments has been documented in organisms across all kingdoms (Bayer-Santos et al., 2014; Lambertz et al., 2015; Peres da Silva et al., 2015; Tsatsaronis et al., 2018; Alves et al., 2019; Tosar and Cayota, 2020; Wang et al., 2021). Full length tRNAs constitute a large part of the RNAs found in EVs in many studies (Nolte-’t Hoen et al., 2012; Zhang et al., 2020; Tosar et al., 2015; Shurtleff et al., 2017). For instance, over-representation of tRNAs has been observed in EVs released by breast cells, bone marrow and adipose-mesenchymal stem cells, lung cells, and EVs from biofluids (Vojtech et al., 2014; Baglio et al., 2015; Tosar et al., 2015). However, different types of EVs may show distinctive, unique cargos. For example, tRNA content differs in EVs shed by melanoma cells, being abundant in microvesicles (EVs: 200–500 μm) and apoptotic bodies but are little represented in exosomes (EVs < 150 μm) (Lunavat et al., 2015). In addition to full length tRNAs, bioactive tRFs are also highly concentrated in EVs (Nolte-’t Hoen et al., 2012; Tosar et al., 2015; Chiou et al., 2018).

Although the major proportion of exRNA does not co-purify with EVs (Arroyo et al., 2011; Tosar et al., 2015), the extra-vesicular compartment of RNA species has just started to receive attention. Studies performed in serum (Dhahbi et al., 2013, 2014) and different types of adherent cultured cells (Tosar et al., 2015) show that fragments derived from ribosomal RNAs (rRNA) and tRNA halves dominate the non-vesicular extracellular compartment, probably forming part of nucleoprotein complexes. Notably, the composition of ex-tRNAs is strongly biased to specific 5′tRFs derived from tRNA^Glu^_CUC_ and tRNA^Gly^_GCC_ (Tosar et al., 2020).

It has been recently shown that the composition of non-vesicular exRNA is largely dependent on their protection from degradation by extracellular nucleases (Nechooshtan et al., 2020; Tosar et al., 2020). This has been demonstrated in a recent study that profiled exRNA in the presence and absence of a ribonuclease inhibitor (Tosar et al., 2020). The ribonuclease inhibitor strongly increased the complexity of exRNA molecules in the cell culture medium, showing highly abundant full length tRNAs and rRNAs that were efficiently cleaved to tRNA- and rRNA-fragments when RNases were not inhibited. Even in the presence of RNases, the abovementioned Glu- and Gly-5′ tRFs remain highly abundant, through the formation of homo- or heterodimeric hybrids that render them resistant to single-stranded RNases. Furthermore, certain tRFs can fold into highly stable intermolecular tetramers stabilized by G-quadruplex structures (Lyons et al., 2017). These data strongly suggest a direct correlation between the abundance of particular exRNA species and their stability.

## The RNA Complexity in Different Types of Extracellular Compartments. Is There Specific Sorting and Release?

Diverse data suggest a specific RNA sorting to different extracellular compartments. For instance, EVs contain miRNAs, full length tRNAs and tRFs, small nucleolar RNAs (snoRNAs), PlWI-interacting RNAs (piRNAs), long non-coding RNAs (lncRNAs), YRNAs, rRNAs, mitochondrial RNAs, and protein-coding RNAs (Bellingham et al., 2012; Crescitelli et al., 2013; Huang et al., 2013; Chakrabortty et al., 2015; van Balkom et al., 2015; Lässer et al., 2017). The HDL cargo, however, contains diverse classes of sRNAs but lacks protein-coding RNAs (Michell et al., 2016), and miRNAs are the principal class binding to extracellular Ago2 (Arroyo et al., 2011; Turchinovich et al., 2011; Michell et al., 2016). It is thus possible that tRNAs and tRFs may also be selectively sorted and released into the extracellular environment to tune the exRNA population for physiological needs.

Extracellular RNA profiles in different extracellular compartments are difficult to compare between studies due to the lack of a consensus experimental approach (Tosar and Cayota, 2020). Methods in EVs isolation differ in the purity of EVs (Monguió-Tortajada et al., 2019), and specific nucleotide species originally assigned to EVs have been recently reassigned to other extracellular compartments (Jeppesen et al., 2019). In addition, separation of RNA in vesicles from RNAs associated with other extracellular carriers, including lipoproteins (Vickers et al., 2011) and ribonucleoproteins (Arroyo et al., 2011; Théry et al., 2018; Mathieu et al., 2019; Murillo et al., 2019) remain challenging, and are still under debate. These technical difficulties are further hampered by the variations in the strategies for RNA purification and library preparation for sequencing purposes, which result in different relative proportions of the multiple classes of RNAs. This is particularly true when attempting to detect and quantify tRNAs and tRFs (Torres et al., 2019).

The comparison between the RNA composition of the source cells and EVs has been used as a readout to demonstrate selective release to the extracellular space. The widely documented asymmetric distribution between these two compartments (Ragusa et al., 2017) agrees with the idea that packaging of exRNAs into EVs is a cell type-dependent, orchestrated process. For instance, studies performed in exosomes released by early passage adipose- and bone marrow-mesenchymal stem cells show that a defined set of miRNAs are overrepresented in exosomes compared to the cell of origin, while other highly expressed miRNAs are precluded from exosomal sorting (Baglio et al., 2015). However, the abundance for the majority of miRNAs correlated in breast cell lines and released exosomes, favoring the idea of a general passive sorting and release (Tosar et al., 2015). In this study, only a few miRNAs showed an asymmetric abundance between cells and exosomes. Some of the extracellular enriched miRNAs were reported as contaminants derived from cell culture additives (Wei et al., 2016; Tosar et al., 2017), which further favors the passive release, at least in breast cell lines.

Just as for other exRNAs, mounting evidence points toward a model where tRNA and tRFs sorting and release can be cell dependent. For example, a selective loading of tRNAs in EVs has been detected in mouse dendritic- and T cells-derived EVs, showing a strong enrichment of the tRNA^Lys^_AAA_, compared to RNA recovered from the intracellular space (Nolte-’t Hoen et al., 2012). Likewise, an uneven proportion of specific tRFs within EVs has been recently reported in T-cells, consistent with a selective export (Chiou et al., 2018). Other studies, however, show a general strong correlation between the cellular and EV content of tRNAs and tRFs in specific adherent cells (Tosar et al., 2015), pointing to a passive sorting and release of these species.

Selective charging of exRNAs into EVs has also been found in bacteria and parasitic eukaryotes. For example, tRFs but not full-length tRNAs can be detected in EVs derived from *Escherichia coli* (Ghosal et al., 2015), while EVs from other bacterial species are enriched in intergenic regions, rRNAs, mRNAs, and other non-coding RNAs (Sjöström et al., 2015; Malabirade et al., 2018). An interest in bacterial exRNAs is emerging given their potential role in bacterial–host interactions (Tosar and Cayota, 2020). Likewise, exRNA biology in host–parasite interaction is gaining attention with reports on protozoan parasites that can release EVs containing, among other RNA species, ex-tRFs that can be taken up by, and exert biological functions in mammalian cells (Garcia-Silva et al., 2014). Finally, the ex-RNA composition of EVs in helminths also seems to be species-specific. While ex-tRFs are depleted in EVs from *Heligmosomoides polygyrus* (Buck et al., 2014), EVs from *Schistosoma mansoni* contain a wide variety of ex-tRFs (Hoy et al., 2014).

The specific sorting into EVs or RBP needs to be distinguished from how release is regulated. This constitutes an additional layer of complexity in understanding the relationship between intracellular RNA and exRNA dynamics. For instance, increased release of EVs following stress or a physiological stimulus has been reported (Basso et al., 2013; Yuana et al., 2013; Chiou et al., 2018; O’Neill et al., 2019); however, the underlying mechanisms are largely unknown. Likewise, the mechanisms by which non-vesicular RNAs are released have not been explored. It has recently been reported an autophagy-dependent mechanism of secretion of cytoplasmic nucleic acids (Jeppesen et al., 2019). However, the composition and abundance of cell RNAs are in good correlation with exRNAs in diverse adherent cells, including the DU145 cell line deficient in autophagy (Tosar et al., 2020), which suggests that other mechanism(s) may be involved in the release on non-EVs exRNA.

Although the exact molecular mechanisms of sorting remain quite elusive, selective charging of exRNAs into EVs has been shown to be mediated by Ago2 and regulated by KRAS-MEK signaling pathway (Cha et al., 2015; McKenzie et al., 2016), and association with other RNA binding proteins (RBP) (Fabbiano et al., 2020) such as ALIX (Iavello et al., 2016) annexin A2 (Hagiwara et al., 2015), major vault protein (MVP) (Teng et al., 2017; Statello et al., 2018), and HuR (Mukherjee et al., 2016). It has also been shown that specific motifs in miRNAs (e.g., GGAG and GGCU) are recognized by the chaperone proteins heterogeneous nuclear ribonucleoprotein A2B1 (hnRNPA2B1), heterogeneous nuclear ribonucleoprotein U (hnRNPU), and RNA-interacting protein SYNCRIP, among others, and selectively sorted into EVs (Villarroya-Beltri et al., 2013; Fabbiano et al., 2020; Zietzer et al., 2020). Another mechanism involves the recognition of the miRNA secondary structure by the RNA-binding protein Y-box I (YBX-1), rather than the primary RNA sequence (Shurtleff et al., 2016). While these studies have been centered in miRNAs, specific motifs recognized by RBP and secondary structures are also present in tRNAs and tRFs, which may similarly facilitate their selective loading into EVs. For example, the precursor tRNA^Ile^_AUA_ can enter the miRNA biogenesis pathway via interactions with Exportin-5, Dicer, and Ago proteins (Hasler et al., 2016). Indeed, processing of tRNAs into tRFs often results in the generation of other classes of small RNAs such as miRNAs or piRNAs (Cole et al., 2009; Keam et al., 2014), which can be released into the extracellular compartment. For instance, the piRNA piR-61648 is a tRF derived from the 5′ arm of tRNA^Gly^_GCC_ and is the most abundant piRNA in human saliva (Ogawa et al., 2013).

Specific tRFs may be actively loaded into EVs and/or generated within vesicles. For instance, angiogenin (ANG), the nuclease responsible for the generation of specific tRFs under stress conditions is present both inside cells and in EVs (Wei et al., 2017). This opens a scenario where specific ANG generated tRFs in EVs can have intra- and extracellular origins.

## Is Capture of Ex-tRNA/tRFs by Recipient Cells Selective?

Extracellular RNA in EVs can be transported into target cells through multiple mechanisms, involving internalization (exosomes) or direct fusion (microvesicles) with the plasma membrane of the target cells. However, the full process involving targeting, entry, and release of the contents into the recipient cell is incompletely characterized (Svensson et al., 2013; Zhou et al., 2016; Mathieu et al., 2019; Zhao et al., 2020).

It seems that capture and internalization of EVs can occur through passive, non-specific endocytosis, or selective receptor mediated endocytosis. Numerous examples in the literature show that exosomes released from particular cells are captured only by specific types of cells, depending on the presence of membrane factors or receptors (Fitzner et al., 2011; Chivet et al., 2014; Mulcahy et al., 2014). Therefore, vesicular delivery of tRNA and tRF molecules could occur through selective mechanisms involving particular membrane receptors recognized by exosomal ligands. However, a recent study shows that the expression levels of a highly stable Gly- tRNA half in MCF-7 cells are strongly correlated with levels in released EVs and in target cells exposed to EVs, thus favoring a passive release and uptake, at least in this paradigm (Gámbaro et al., 2020).

Non-vesicular exRNA transport into target cells has been shown for miRNAs associated with HDL, depending on a specific receptor (scavenger receptor class B type 1) (Vickers et al., 2011). This opens the likely possibility that tRNAs/tRFs complexed with HDL, which are by far more abundant than miRNAs (Tosar et al., 2020), analogously enter target cells. Uptake of extracellular, vesicular free miRNAs complexed to Ago2 has been shown to occur through binding to Neuropilin-1, which acts as a receptor of this ribonucleoprotein complex (Prud’homme et al., 2016). Selected tRNAs and tRFs have been shown to interact with Ago proteins (Woolnough et al., 2015; Pong and Gullerova, 2018), suggesting a comparable capture and internalization process. A number of extracellular RBP exist that may provide analogous scenarios for protection of tRNAs and tRFs, making them potential signaling molecules.

Naked RNA is also captured by cells *in vivo*. For instance, extracellular dsRNAs are internalized by cells via clathrin- (Itoh et al., 2008) and raftlin-dependent endocytosis (Watanabe et al., 2011). In addition, the receptor for advanced glycation end-products (RAGE) that is expressed on the cell surface binds to RNA in a sequence-independent manner and enhances uptake into endosomes (Bertheloot et al., 2016). RAGE internalized RNAs increases the sensitivity to single stranded RNA-sensing Toll-like receptors (TLRs). TLRs localize to the cell surface and/or to endosomes and different TLR types show preferential recognition of ssRNA or dsRNAs, with sequence and/or structural specificities (Liu and Gack, 2020). For instance, TLR3 is expressed in the plasma membrane of fibroblasts and epithelial cells, and is able to sense extracellular, naked RNA species (Alexopoulou et al., 2001; Matsumoto et al., 2002). Although the mechanism is uncertain, spontaneous uptake of Ala- and Cys-5′ tRNA halves has been shown (Ivanov et al., 2014). Recent studies have shown that the 3′ CCACCA sequence of tRNA^Ala^_UGC_ can be effectively recognized by TLR3 in HEK293 cells and induce immune response (Wang et al., 2006). Because naked double-stranded tRF heterodimers are so abundant extracellular components, it is tempting to speculate about the possibility that these species activate TLR specifically sensing dsRNAs.

We hypothesize that specific, yet to be identified receptors may exist for ex-tRNA/ex-tRFs free or bound to different RBP with the capacity to internalize these species. Both the carrier (RBP) and the receptor may provide layers of specificity in ex-tRNA/ex-tRFs binding and uptake.

## Possible Functions of Ex-tRNA/Ex-tRFs: Do They Reflect Trna Metabolism in Producing Cells And/Or Exert a Paracrine Activity?

RNA release to the extracellular compartment does not necessarily involve a functional role of all species as paracrine mediators. However, the high abundance of ex-tRNA/ex-tRFs, with independence on whether they are passively or actively released, favors the idea of an active role of these species in cell-to-cell communication. An extreme case in point is shown for tRFs that can enter the maturing sperm through epididymosomes that are secreted from somatic cells in the epididymis (Conine et al., 2018), and have been shown to contribute to intergenerational inheritance (Chen et al., 2016).

Within cells, captured tRFs may trigger a number of signaling cascades that have been validated in cell cultures and/or biochemical approaches, including regulation of gene expression, protein translation, and response to stress (Magee and Rigoutsos, 2020). Beyond the studies on the effects of tRFs delivered through transfection reagents, increasing evidence suggests a physiological interaction of tRFs with RNA sensors that modulate the innate immune response, as discussed above.

Importantly, even upon efficient cellular uptake, not all internalized exRNAs may be functionally relevant. A major challenge will be to accurately assess the degree of productive and non-productive cellular uptake within recipient cells. For example, endosomal entrapment is a major issue in RNA therapeutics (Crooke et al., 2017). It is possible that internalized ex-tRNA/ex-tRF could face a similar fate, thus limiting their functional potential. Because the mechanisms of cellular uptake could influence their intracellular trafficking (Juliano et al., 2014), we propose that ex-tRNA/ex-tRNA function should be evaluated in the context of their mechanisms of delivery, uptake, and intracellular trafficking.

## A Role for tRNA Modifications in Ex-tRNA/Ex-tRF Biology?

tRNAs are the most heavily post-transcriptionally modified nucleic acids in the cell. To date, more than 150 different chemical modifications have been found in tRNAs (Boccaletto et al., 2018), where they play important roles in tRNA structure, stability, and function (Pan, 2018). It is thus possible that tRNA modifications may also be involved in regulating ex-tRNA/ex-tRF generation, stability, uptake, and function.

tRNA modifications can protect tRNAs and tRFs from cleavage or degradation. For instance, 5-methylcytosine (m5C) that is present in several tRNAs can prevent tRNA cleavage by ANG and modulate tRNA processing into tRFs (Tuorto et al., 2012; Blanco et al., 2014). Likewise, 2′-*O*-methylated (2′-OMe) nucleotides, which are abundant in tRNAs, are usually not recognized by RNases and are frequently used in RNA therapeutics to prevent oligonucleotide degradation (Khvorova and Watts, 2017). In an analogous manner, these modified residues could be protecting tRNAs and tRFs from nucleolytic cleavage (Oberbauer and Schaefer, 2018). In addition, chemical modifications such as 2′-OMe increase the melting temperature of RNA:RNA duplexes (Majlessi et al., 1998). It is plausible that in the context of ex-tRF stability, such modifications may not only be maintaining ex-tRF integrity but also be promoting ex-tRF duplex formation.

As discussed above, naked RNA can be recognized by cell surface receptors and be internalized. We speculate that tRNA modifications can also affect cellular uptake of ex-tRNA/ex-tRFs. Interestingly, patterns of RNA modification can serve as the basis for discrimination between self and non-self RNAs. Bacterial tRNAs were shown to stimulate the innate immune system via TLR7, and a 2′-OMe modification at position 18 of bacterial tRNAs was sufficient to abolish this immunostimulatory effect (Jöckel et al., 2012). We note that TLR7 is not localized to the cell surface, but the abovementioned example indicates that tRNA modifications can alter receptor-mediated tRNA recognition. Indeed, different modifications in other classes of RNAs have also been shown to abolish signaling through TLRs (Karikó et al., 2005). Furthermore, extracellular single-stranded inosine-containing RNAs are internalized in epithelial cells by scavenger receptor class-A-mediated endocytosis (Liao et al., 2011). Inosine is commonly found at position 34 of tRNAs (Torres et al., 2014b, Torres et al., 2015), and can induce cleavage of tRNAs mediated by Endonuclease V (Morita et al., 2013; Vik et al., 2013). Thus, inosine may represent an example of a tRNA modification that could regulate both tRF formation and ex-tRF internalization.

Lastly, tRNA modifications can also serve as recognition elements for RBPs. For example, several tRNA modifications are important for their efficient recognition by aminoacyl tRNA synthetases (that charge tRNAs with their appropriate amino acid), while others such as 1-methyladenosine at position 58 of tRNAs modulate tRNA affinity for the elongation factor 1A (that delivers tRNAs to the ribosomal A-site for protein synthesis) (Pan, 2018). In the context of extravesicular ex-tRNA/ex-tRFs, one could imagine that tRNA modifications could modulate ex-tRNA/ex-tRF binding to potential partners. Sera of patients suffering from myositis contain autoantibodies of the anti-PL-12 type that interact with the anticodon loop of human tRNA^Ala^_AGC_. Interestingly, two modifications present in this tRNA (inosine at position 34 and *N*^1^-methylinosine at position 37 in the anticodon loop) are major epitopes for these autoantibodies (Becker et al., 1999). This suggests that tRNA modifications within ex-tRNAs may play a role in the interaction of such ex-tRNAs with the immune system. In addition, tRNA modifications may at least be important for ex-tRNA/ex-tRF function within recipient cells.

## Discussion

tRNAs have received strong attention in the recent years with new assigned functions besides the canonical role as genetic code decoders in protein translation. Their participation as modulators of translation (Wilusz, 2015) and adaptation to stress (Kirchner and Ignatova, 2015) is complemented with the finding that tRNAs can be actively processed to smaller, bioactive fragments (Magee and Rigoutsos, 2020). Specific types of tRFs cannot be envisioned as by-products of tRNA turnover and mounting evidence reveal them as a novel class of sRNAs that regulate gene expression through multiple mechanisms.

The regulatory activities of tRNAs and tRFs modulate cell signaling and are correlated with human disease. For instance, increased levels of specific tRNAs promote metastatic progression by inducing the expression of particular proteins (Goodarzi et al., 2016). tRF expression dynamics also participate in disease processes, through the regulation of apoptosis, protein synthesis, and/or RNA interference (Soares and Santos, 2017). In addition, tRNA/tRF activity is modulated by tRNA modifications, thus defective tRNA modifications have also been associated to complex diseases such as cancer, type 2 diabetes, and neurological disorders (Torres et al., 2014a).

The complexity of tRNAs/tRFs biology, physiology, and pathology is further magnified by their release to the extracellular space, depending on the status of the cell of origin. Emerging evidence suggests that tRNA and tRFs are among the most abundant species in biofluids and in cell culture media, both within EVs and especially outside EVs. Although many studies point toward a regulated sorting and release of tRNA/tRFs, the data presented here suggest that the complexity of the non-vesicular ex-tRNAs/ex-tRFs does not necessarily correlate with a differential release. Instead, it may reflect an interplay between the intracellular RNA composition, its passive and non-passive extrusion, and the abundance and types of extracellular RNases.

In thinking of a productive effect of particular types of exRNAs in target cells, the number of internalized molecules is an important factor. It has been suggested that the few copies of miRNAs in biofluid exosomes make them unlikely molecules activating signaling in target cells (Chevillet et al., 2014). However, the strong abundance of tRNAs and tRFs in the extracellular compartment points them as likely actors in cell-to-cell communication. It has been recently shown that the overexpression of tRNA halves resistant to degradation is sufficient to trigger their encapsulation into EVs and delivery to target cells (Gámbaro et al., 2020). Because stable tRNA halves are highly produced in cells under stress conditions through the activity of ANG (Yamasaki et al., 2009), it is easy to imagine that upon release, recipient cells could sense these types of tRFs as a sign of stress. For non-vesicular tRFs more exposed to RNases, stability may be especially relevant for productive signaling in target cells. While the less stable tRNAs/tRFs have opportunities to target cells nearby, the more stable and abundant tRFs may induce signaling at more distant places.

With independence on the mechanism of release, stability, and possible biological activity, it is worth mentioning that the high abundance of tRFs in biofluids has opened an active field of research in biomarker discovery. The biogenesis of stress linked tRFs is reflected in plasma, with altered profiles in diseases such as cancer (Dhahbi et al., 2014; Wang et al., 2020) or epilepsy (Hogg et al., 2019). This highlights the need to include these species in the definition of prognostic and/or diagnostic biosignatures.

In summary, the significance of ex-tRNA/tRFs is an exciting emerging field in functional biology and translational medicine. Future research needs to expand on the mechanistic basis of (1) tRNA and tRFs sorting into EVs and non-EVs compartments, (2) the regulation of passive versus selective tRNA/tRFs release, and (3) uptake by target cells. The complexity of these studies is enhanced by cell type-specific processes, the influence of highly abundant tRNA modifications, and the need to overcome a number of technical issues to reliably characterize exRNAs.

## Data Availability Statement

The original contributions presented in the study are included in the article. Further inquiries can be directed to the corresponding author/s.

## Author Contributions

AT and EM contributed to the conception of the manuscript and wrote all sections. All authors contributed to the article and approved the submitted version.

## Conflict of Interest

The authors declare that the research was conducted in the absence of any commercial or financial relationships that could be construed as a potential conflict of interest.
